# Gray matter density reduction associated with adjuvant chemotherapy in older women with breast cancer

**DOI:** 10.1007/s10549-018-4911-y

**Published:** 2018-08-07

**Authors:** Bihong T. Chen, Taihao Jin, Sunita K. Patel, Ningrong Ye, Can-Lan Sun, Huiyan Ma, Russell C. Rockne, James C. Root, Andrew J. Saykin, Tim A. Ahles, Andrei I. Holodny, Neal Prakash, Joanne Mortimer, James Waisman, Yuan Yuan, Daneng Li, George Somlo, Jessica Vazquez, Abrahm Levi, Heidi Tan, Richard Yang, Vani Katheria, Arti Hurria

**Affiliations:** 1 Department of Diagnostic Radiology, City of Hope National Medical Center, 1500 East Duarte Road, Duarte, CA 91010 USA; 2 Center for Cancer and Aging, City of Hope National Medical Center, Duarte, CA USA; 3 Department of Population Science, City of Hope National Medical Center, Duarte, CA USA; 4 Division of Mathematical Oncology, City of Hope National Medical Center, Duarte, CA USA; 5 Neurocognitive Research Lab, Memorial Sloan Kettering Cancer Center, New York, NY USA; 6 Center for Neuroimaging, Indiana University School of Medicine, Indianapolis, IN USA; 7 Department of Radiology, Memorial Sloan-Kettering Cancer Center, New York, NY USA; 8 Division of Neurology, City of Hope National Medical Center, Duarte, CA USA; 9 Department of Medical Oncology, City of Hope National Medical Center, Duarte, CA USA

**Keywords:** Breast cancer, Chemotherapy, Cognition, Gray matter density (GMD), Voxel-based morphometry (VBM)

## Abstract

**Purpose:**

The purpose of this study was to evaluate longitudinal changes in brain gray matter density (GMD) before and after adjuvant chemotherapy in older women with breast cancer.

**Methods:**

We recruited 16 women aged ≥ 60 years with stage I–III breast cancers receiving adjuvant chemotherapy (CT) and 15 age- and sex-matched healthy controls (HC). The CT group underwent brain MRI and the NIH Toolbox for Cognition testing prior to adjuvant chemotherapy (time point 1, TP1) and within 1 month after chemotherapy (time point 2, TP2). The HC group underwent the same assessments at matched intervals. GMD was evaluated with the voxel-based morphometry.

**Results:**

The mean age was 67 years in the CT group and 68.5 years in the HC group. There was significant GMD reduction within the chemotherapy group from TP1 to TP2. Compared to the HC group, the CT group displayed statistically significantly greater GMD reductions from TP1 to TP2 in the brain regions involving the left anterior cingulate gyrus, right insula, and left middle temporal gyrus (*p*_FWE(family-wise error)-corrected_ < 0.05). The baseline GMD in left insula was positively correlated with the baseline list-sorting working memory score in the HC group (*p*_FWE-corrected_ < 0.05). No correlation was observed for the changes in GMD with the changes in cognitive testing scores from TP1 to TP2 (*p*_FWE-corrected_ < 0.05).

**Conclusions:**

Our findings indicate that GMD reductions were associated with adjuvant chemotherapy in older women with breast cancer. Future studies are needed to understand the clinical significance of the neuroimaging findings. This study is registered on ClinicalTrials.gov (NCT01992432).

## Background

Patients who are receiving adjuvant chemotherapy for breast cancer have reported cognitive changes associated with receipt of cancer therapy [1]. However, the biologic basis for the cognitive issues is poorly understood. Prior studies in women with breast cancer have observed brain structural alterations with exposure to chemotherapy [2, 3]. Few studies have focused on older women over 60 years of age who represent almost half of the new breast cancers diagnosed in the United States [4].

Neuroimaging studies have observed a decrease in gray matter density (GMD) and working memory function in younger patients with breast cancer with exposure to chemotherapy at mean age of 46.3 (SD = 6.1) years to 52.9 (SD = 8.6) years [5–7]. Longitudinal studies have shown acutely reduced GMD 1 month after treatment in younger patients with breast cancer with mean ages at early 50 years of age [6, 8]. However, none of these previous studies has specifically focused on older women with breast cancer. Moreover, there is a lack of assessment of neural correlates in older women with breast cancer who have had a history of exposure to chemotherapy.

In order to access the potential adverse impact of cancer treatments on brain structure and function in older patients receiving adjuvant chemotherapy, we performed a pilot study to evaluate longitudinal changes in GMD utilizing brain MRI scans. We hypothesized that GMD would be reduced in older women with breast cancer from pre- to post-adjuvant chemotherapy and the changes in GMD may be associated with a detrimental effect on cognitive performance.

## Materials and methods

### Participants

This was a longitudinal study of breast cancer patients scheduled to receive adjuvant chemotherapy and age-/sex-matched healthy controls. The details of the study have been reported previously [9]. The eligibility criteria for patients with breast cancer were the following: diagnosis of stage I–III breast cancer, age 60 years and older, scheduled to receive adjuvant chemotherapy. The patients were excluded if they had metastatic disease or contraindications for brain MRI scan such as cardiac pacemaker, orbital metal implants or claustrophobia. Age- and sex-matched healthy controls with no cancer history or prior exposures to chemotherapy were recruited from the community through advertisement in local newspapers, patients’ referral of friends and family, and health fairs.

This research protocol was approved by the Institutional Review Board at City of Hope National Medical Center. Informed consent was obtained from all study participants.

Study measures including brain MRI scans and NIH Toolbox for Cognition testing were completed both upon enrollment at baseline (time point 1, TP1) and within 1 month following the completion of chemotherapy (time point 2, TP2). Healthy controls underwent the same assessments at matched intervals as the chemotherapy group.

### Brain MRI acquisition

All participants underwent brain MRI scans at both time points on the same 3T Verio Siemens scanner (Siemens, Erlangen, Germany). The details of brain MRI imaging protocol have been described previously [9]. Briefly, both sagittal and axial T1-weighted three-dimensional (3D) magnetization prepared rapid gradient echo (MPRAGE) imaging data were acquired. A 3D fluid-attenuated inversion recovery (FLAIR) sequence was obtained to rule out incidental brain pathology.

### Neuroimaging analysis

GMD was evaluated with the voxel-based morphometry (VBM) approach using the Diffeomorphic Anatomical Registration Through Exponentiated Lie Algebra (DARTEL) Toolbox in the Statistical Parametric Mapping software version 12 (SPM 12) (Wellcome Trust Centre for Neuroimaging, London, UK). It was performed using the following steps: (1) segmenting each T1 weighted image to generate the tissue (gray matter, white matter, and cerebrospinal fluid) probability maps; (2) using DARTEL tool to improve the inter-subject alignment [10]; (3) spatially normalizing the gray matter probability maps into Montreal Neurological Institute (MNI) space [11] and smoothing (FHWM = 10 mm). The gray matter probability maps computed from axial and sagittal T1 images for each scan were averaged after spatially normalized and smoothed for the group level statistical analysis. All T1 images are manually adjusted to reset the origin at the anterior commissural level and to set the posterior commissural level for the second axis of the coordinate system.

### NIH toolbox for cognition testing

All study participants were administered a neuropsychological testing battery using the NIH Toolbox for Cognition [12]. Using normative data from a large national standardization sample as reference, the NIH Toolbox for Cognition employed measures to evaluate several subdomains such as working memory, attention, executive function, processing speed, episodic memory, and language. It took approximately 30 min to perform this battery which generated a total of 10 scores consisting of 3 composite scores and 7 individual scores.

### Statistics analysis

The group differences in frequency distributions of baseline demographic characteristics of the participants were assessed using Fisher’s exact tests. The group differences in the means of baseline age were assessed using a two-sample student *t* test. We considered a two-sided *p* value less than 0.05 as statistically significant.

The GMD changes between two points in the healthy control (HC) and the chemotherapy (CT) groups, as well as the group difference in the GMD changes, were assessed using a mixed-design repeated measurement two-way Analysis of Variance (ANOVA) model in SPM12. The model included the group factor (HC and CT), the time factor (TP1 and TP2), and the subject factor that was used to account for the subject effect in repeated measurements. The analysis was implemented using the flexible factorial design in SPM12. We presented data for the summary of all significant clusters (*p*_corrected_ < 0.05) identified in voxel-wise ANOVA analysis of GMD. It includes cluster extent, cluster level *p* values, MNI coordinates, family-wise error rate corrected voxel level *p* value (*p*_FWE-corrected_), and anatomical regions of the cluster peaks. Local peaks in the clusters were included if the voxel level *p*_FWE-corrected_ values were smaller than 0.05.

We performed voxel-wise regression analysis to explore potential linear correlations between baseline GMD and testing scores from the NIH Toolbox for Cognition in the brain regions where the CT group displayed statistically significant greater GMD reduction than the HC group. We also explored potential linear correlations in the CT group between GMD change and the changes in the neuropsychological testing scores before and after chemotherapy, using the same type of analysis. We used the baseline GMD as the independent variable and the baseline testing scores from the NIH Toolbox for Cognition as the regressor. When exploring potential linear correlations between two change scores, these baseline measurements were changed to their corresponding change scores between TP1 and TP2. The total intracranial volume (ICV) and the age of the subjects were used as covariates in all analysis to control for the effects of these two variables on GMD. The ICV was computed by summing the probabilities of gray matter, white matter and cerebrospinal fluid. The results were visualized using the xjView tool (http://www.alivelearn.net/xjview). The statistical significance-based region of interest (ROI) was identified according to the family-wise error rate adjusted voxel level *p* value of the cluster peaks (*p*_FWE-corrected_ ≤ 0.05). Unadjusted *p* value threshold (*p* ≤ 0.001) was used for building clusters.

## Results

### Characteristics of participants

Table 1 shows the characteristics of participants enrolled in this analysis. Sixteen female patients with breast cancer (mean age 67 years, range 60–82 years) and 15 age-matched healthy controls (mean age: 68.5 years, range 60–78 years) completed study measures for both time points. There was no significant difference in age (*t* test *p* = 0.37) or education (Fisher’s exact test *p* = 0.58) between the CT group and the HC group. The CT group included 69% white females and 31% black females, whereas HC group consisted of all white females (Fisher’s exact test *p* = 0.04). CT group included 31% patients with stage I, 50% patients with stage II and 19% patients with stage III breast cancer. Forty-four percent patients received docetaxel and cyclophosphamide; 25% patients received paclitaxel and trastuzumab; and the remaining 32% (5 patients) received 5 different chemotherapy regimens.

**Table 1:** Demographic/disease/treatment information of the study participants

Variable	Chemotherapy (CT)	Healthy control (HC)
No. of participants	16	15
Age (years)		
Mean	67.0	68.5
SD	5.39	5.69
Range	60–82	60–78
Race		
White	11 (69%)	15 (100.0%)
Black	5 (31%)	0 (0.0%)
Education		
High school	4 (25.0%)	1 (6.7%)
Some college	8 (50.0%)	8 (53.3%)
College degree	3 (18.8%)	4 (26.7%)
Post college	1 (6.3%)	2 (13.3%)
Stage		
I	5 (31.3%)	N/A
II	8 (50.0%)	N/A
III	3 (18.8%)	N/A
Regimen		
TC	7 (43.8%)	N/A
TCHP	1 (6.3%)	N/A
Paclitaxel/trastuzumab	4 (25%)	N/A
TCyHP	1 (6.3%)	N/A
Carboplatin/paclitaxel	1 (6.3%)	N/A
ddAC followed by paclitaxel	1 (6.3%)	N/A
TAC	1 (6.3%)	N/A

*TC* docetaxel and cyclophosphamide, *TCHP* docetaxel, carboplatin, trastuzumab, pertuzumab, *TCyHP* docetaxel, cyclophosphamide, trastuzumab, pertuzumab, *ddAC followed by paclitaxel* dose-dense adriamycin cyclophosphamide followed by paclitaxel, *TAC* docetaxel, doxorubicin, and cyclophosphamide

### GMD measurement

#### Baseline GMD

There were no brain regions showing statistically significant between-group differences in GMD cross-sectionally at baseline after correction for the multiple comparisons (*p*_corrected_ > 0.05).

#### Within-group GMD changes between TP1 and TP2

There were substantial GMD reductions in the CT group from TP1 to TP2 (*p*_corrected_ < 0.05) (Table 2). The affected brain regions included bilateral inferior frontal gyri, bilateral insula, left anterior cingulate, left inferior frontal gyrus (BA 47), left middle temporal gyrus, left caudate and right middle frontal gyrus (Table 2). In the HC group from TP1 to TP2, no gray matter density reductions were statistically significant (*p*_FWE-corrected_ > 0.05).

**Table 2:** Regional gray matter density alterations

MNI coordinates (x y z)^a^	Cluster extent (k)	Cluster-level	Voxel-level	T	Region (selected local maxima^b^)
		*p*_corrected_	*p*_FWE-corrected_		
Within group					
Chemotherapy: TP2 < TP1					
− 43.5 24 − 1.5	109,650	< 0.0001	0.0003	8.86	L Inferior Frontal Gyrus
37.5 24 9	109,650	< 0.0001	0.0097	7.14	R Inferior Frontal Gyrus
39 − 19.5 18	109,650	< 0.0001	0.0006	8.47	R Insula
− 39 − 15 16.5	109,650	< 0.0001	0.0008	8.3	L Insula
− 7.5 34.5 27	109,650	< 0.0001	0.0009	8.28	L Anterior Cingulate
− 37.5 18 − 3	109,650	< 0.0001	0.0024	7.78	L Inferior Frontal Gyrus (BA 47)
− 48 − 31.5 − 4.5	109,650	< 0.0001	0.0047	7.48	L Middle Temporal Gyrus
− 7.5 12 0	2381	< 0.0001	0.1788	5.77	L Caudate (Caudate Head)
31.5 − 4.5 49.5	514	0.0012	0.2267	5.65	R Middle Frontal Gyrus
Between-group					
Reductions in chemotherapy > healthy control					
− 7.5 34.5 27	1591	< 0.0001	0.015	6.94	L Anterior Cingulate
39 − 19.5 18	3571	< 0.0001	0.027	6.67	R Insula
− 39 − 15 16.5	4315	< 0.0001	0.0847	6.14	L Insula
− 64.5 − 25.5 − 7.5	2785	< 0.0001	0.0448	6.43	L Middle Temporal Gyrus
− 19.5 − 46.5 − 9	9242	< 0.0001	0.0613	6.29	L Parahippocampal Gyrus
24 − 63 − 14	6444	< 0.0001	0.036	5.95	R Fusiform Gyrus
43.5 43.5 12	462	0.0025	0.4189	5.31	R Inferior Frontal Gyrus (BA 46)
40.5 − 45 36	930	< 0.0001	0.6784	4.97	R Supramarginal Gyrus (BA 40)
58.5 − 22.5 − 7.5	508	0.0013	0.7433	4.89	R Middle Temporal Gyrus (BA 21)

*TP1* time point 1, *TP2* time point 2, *L* left, *R* right, *FWE* family-wise error

^a^MNI coordinates of the cluster peaks and those local peaks within each cluster whose voxel-level family error rate corrected p values smaller than 0.001 were listed in the table

^b^The anatomical regions of the peaks were indicated according to the Talairach Daemon atlas

#### Between-group comparison of GMD changes

**Fig. 1:**
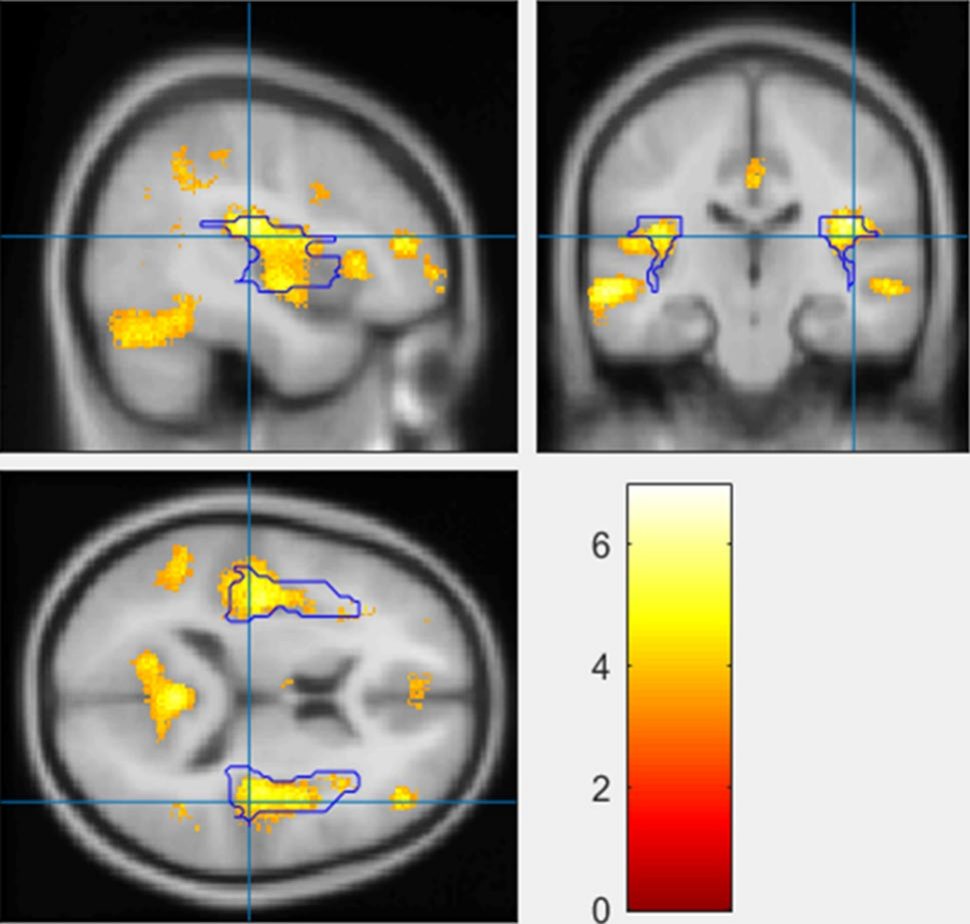
Highlighted brain regions indicating greater gray matter density (GMD) reduction in the chemotherapy group than in the healthy control group

There were 9 clusters where the CT group displayed significantly greater reductions than the HC group in GMD at *p*_corrected_ < 0.05 (Table 2; Fig. 1). Some clusters were distributed in a bilateral pattern in the brain. The peaks of the clusters were located in left anterior cingulate, bilateral insula, left middle temporal gyrus, left parahippocampal gyrus, right fusiform gyrus, right inferior frontal gyrus (BA 46), right supramarginal gyrus (BA 40), and right middle temporal gyrus (BA 21). In addition, three of the clusters located in left anterior cingulate gyrus, right insula, and left middle temporal gyrus also reached statistical significance at the voxel level (*p*_FWE-corrected_ < 0.05).

### Neuropsychological testing

The summary of all NIH Toolbox testing scores for this study has been reported previously [9]. Briefly, there were no significant differences between the CT group and the HC group in the NIH Toolbox for Cognition testing scores at TP1 or at TP2, or the changes in the testing scores from TP1 to TP2 (*p* values > 0.05).

### Correlative analysis between GMD and neuropsychological testing scores

**Fig. 2:**
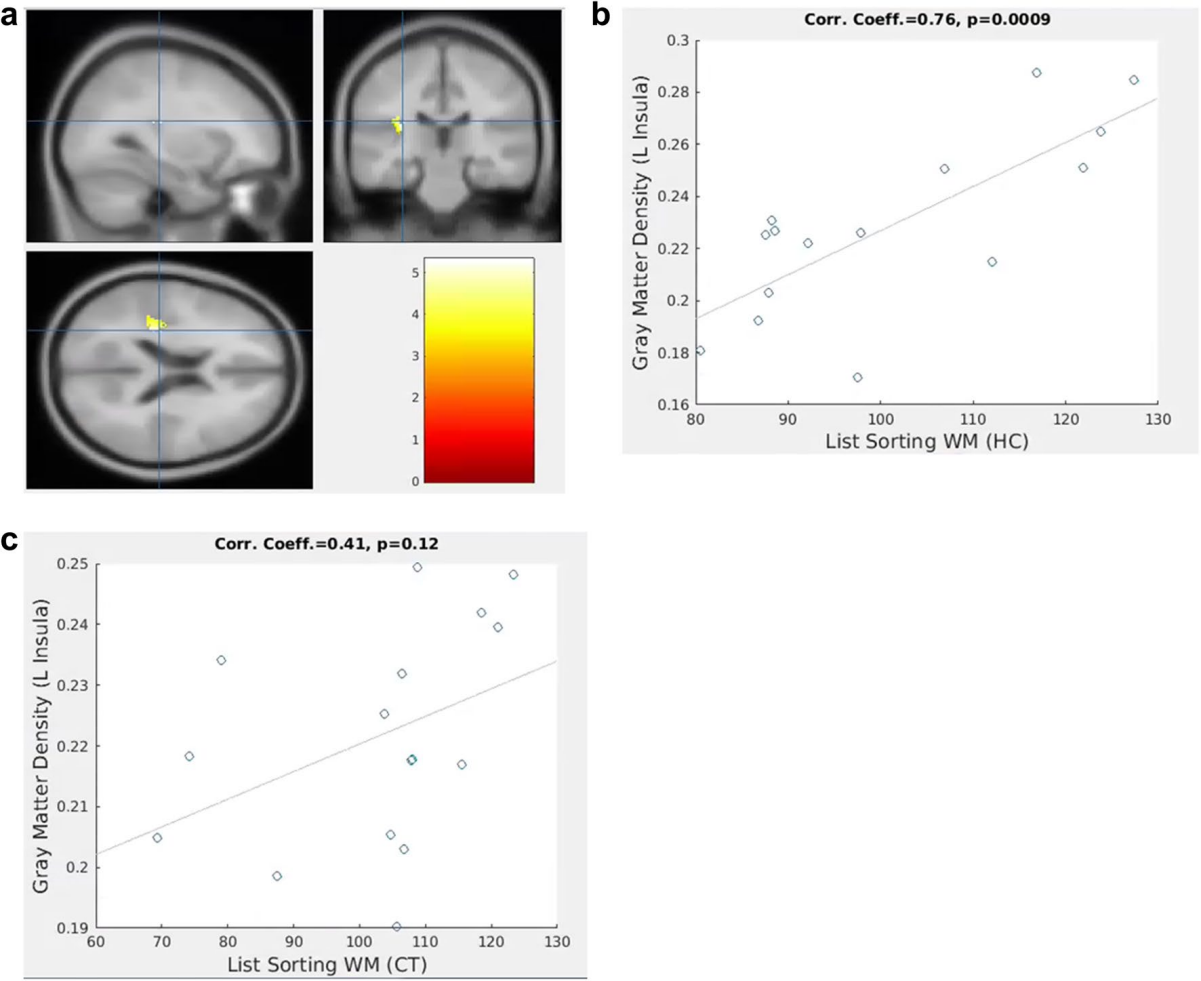
Correlation between baseline gray matter density (GMD) in left insula and list-sorting working memory scores. **a** Brain region in left insula at MNI coordinates (− 30 − 26 20) where baseline GMD in the healthy control group (HC) was significantly correlated with list-sorting working memory score. **b** Significant correlation between baseline GMD in left insula and list-sorting working memory score in the HC group (correlation coefficient = 0.76, *p* = 0.0009). **c** Correlation between baseline GMD in left insular and list-sorting working memory score in the chemotherapy group (CT) (correlation coefficient = 0.41, *p* = 0.12)

Our voxel-wise linear regression analysis detected a 240-voxel size cluster (Fig. 2a) where the baseline GMD was statistically significantly correlated with the list-sorting working memory score (*p*_FWE-corrected_ < 0.05). The cluster peak of the t-statistic was located in the left insula at the MNI coordinates (− 30 − 26 20). Furthermore, correlative analysis of the baseline GMD at the cluster peak and the list-sorting working memory score confirmed a strong positive correlation in the HC group (correlation coefficient = 0.76, *p* = 0.0009, Fig. 2b). There was also a trend of correlation in the CT group with correlation coefficient of 0.41, although it was not statistically significant (Fig. 2c, *p* = 0.12). None of the other baseline NIH Toolbox cognitive testing scores was significantly correlated with baseline GMD (*p*_FWE-corrected_ > 0.05).

Voxel-wise regression analysis did not detect any significant linear correlation between GMD changes and the changes in all the neuropsychological testing scores in the CT group (*p*_FWE-corrected_ > 0.05).

## Discussion

Our study showed acute GMD reduction in older patients with breast cancer from pre- to post- exposure to adjuvant chemotherapy. To the best of our knowledge, the present study is one of the few longitudinal studies focusing on the potential adverse impact of adjuvant chemotherapy on brain structure and function in older patients.

Our study findings of GMD reduction in older patients with breast cancer who had received chemotherapy are consistent with findings of prior studies in younger patients [13]. Furthermore, the affected brain regions in our study were in general agreement with prior neuroimaging literature [14]. Prior longitudinal studies with similar study design but in a younger patient population showed acute reduction in frontal temporal GMD at 1 month after chemotherapy and the frontal temporal lobes were the brain regions most sensitive to the chemotherapy insult [5–7]. Our study with a focus on older adults with cancer has also identified brain regions in the frontal temporal lobes that displayed significant GMD reduction including the inferior frontal gyrus and middle frontal gyrus in the chemotherapy-treated group, similar to the affected brain regions identified in the younger population. In addition, we have observed greater GMD reductions in the chemotherapy group as compared to the healthy control group. The involved brain regions included the left anterior cingulate gyrus, bilateral insula, bilateral middle temporal gyri, and left parahippocampal gyrus. Most  of the invovled regions have also been reported previously in other studies with similar longitudinal design in patients with breast cancer with exposure to chemotherapy [6, 8]. Our study identified the frontal temporal brain regions that are known to be critical for executive function such as working memory [7, 15, 16].

In contrast to the prior literature, we also identified GMD reductions in the brain regions that were not previously reported. For example, we identified statistically significant GMD reductions in both the left and the right insular cortex, while a study by Lepage and colleagues showed GMD reduction in the left insula only [8]. In addition, our study detected a greater spatial extent of GMD reduction (manifested by larger cluster sizes) in the affected brain regions than what has been reported by the previous research [6–8]. It is intriguing that our study results have indicated more extensive GMD changes in terms of larger and additional affected brain regions. It is conceivable that our study cohort of older women with breast cancer might be more susceptible to treatment related brain structural alterations. However, it is also possible that a number of other possibilities such as difference in chemotherapy regimens or pre-existing co-morbidities could have contributed to this finding. Further studies with larger sample size are needed to understand what risk factors contribute to more extensive acute GMD reduction in older patients with breast cancer receiving adjuvant chemotherapy.

Although we observed GMD reduction, we did not find a statistically significant positive linear correlation between the GMD reduction and the changes in the neuropsychological testing scores. Other studies in contrast have identified correlation between GMD reduction and neuropsychological testing scores in patients treated with chemotherapy. In particular, a study by Lepage and colleagues observed significant correlation between gray matter reduction in the left insular cortex and processing speed in the chemotherapy-treated group [8]. Another study showed decreased activation in the insula during verbal memory recall testing in patients with breast cancer with exposure to chemotherapy [17]. There are several possible reasons why we did not identify changes in neuropsychological testing. The patients may be able to compensate their brain function to keep their cognitive performance within the norm even though they have decreased GMD [18, 19]. Prior research has shown that patients may enhance their cognitive function by recruiting and activating additional neurocircuitry during a high-memory task load before chemotherapy compared to healthy controls [20]. We speculated that the chemotherapy-treated older patients in our study cohort may have utilized similar compensatory mechanisms; however, our pilot study cannot ascertain the exact reason for our lack of decline in neuropsychological testing. We acknowledge that our modest sample size might not have been adequate enough to identify small differences in cognitive performance.

It is noteworthy that among our patients, the baseline GMD in left insula was positively correlated with the baseline list-sorting working memory score, though the correlation reached statistical significance only in the healthy control group. Our study result provides some credence to our hypothesis that there is an association between neural correlates and neuropsychological testing. Furthermore, our study result of baseline GMD being correlated with cognitive testing was consistent with the previous reports [8, 17, 18]. However, we hypothesize that larger studies will be needed to identify significant correlation between GMD and neuropsychological testing scores in the chemotherapy group.

There were several limitations in this study. First, our study participants were followed longitudinally for a short time course from pre- to post-chemotherapy. Hence, we could not comment on long-term or delayed effect of chemotherapy on brain structure. Second, the patients in our study received a variety of different chemotherapy regimens, which may have caused inhomogeneous changes in GMD. We did not have a sufficient sample size to identify the potentially different effects of chemotherapy regimens. Third, this study did not include a breast cancer control group with no history of chemotherapy, and we could not ascertain definitively whether the findings were attributable to chemotherapy or to a breast cancer diagnosis in general. Lastly, as mentioned above, our sample size was modest, which may have limited the ability to identify small changes in neuropsychological testing or GMD.

Despite the limitations, there were strengths in this study. The prospective longitudinal design of our study allowed assessment of brain structural alteration over time. With a focus on older adults with cancer, we contributed to filling the knowledge gap regarding potential neural correlates of cognition in the older patients with cancer undergoing systemic treatment. This pilot study has served to generate hypotheses for future larger studies to help unravel the biological mechanism responsible for cancer-related cognitive impairment.

## Conclusion

Our findings indicate that GMD reductions were associated with adjuvant chemotherapy in older women with breast cancer. Future larger studies with greater statistical power and independent replication are needed to understand the clinical significance of these neuroimaging findings.
